# Mutational Analysis of Substrate Recognition in Trypsin-like Protease Cocoonase: Protein Memory Induced by Alterations in Substrate-Binding Site

**DOI:** 10.3390/molecules29225476

**Published:** 2024-11-20

**Authors:** Nana Sakata, Shigeru Shimamoto, Yuri Murakami, Orika Ashida, Toshiki Takei, Mitsuhiro Miyazawa, Yuji Hidaka

**Affiliations:** 1 Faculty of Science and Engineering, Kindai University, 3-4-1 Kowakae, Higashi-Osaka 577-8502, Japan; nsakata@life.kindai.ac.jp (N.S.);; 2 Institute of Protein Research, Osaka University, 3-2 Yamada-oka, Suita 565-0871, Japan; 3 Institute of Agrobiological Sciences, National Agriculture and Food Research Organization, Tsukuba 305-8634, Japan

**Keywords:** chaperone, chymotrypsin, cocoonase, folding, substrate binding

## Abstract

To investigate the substrate recognition mechanism of trypsin-like protease cocoonase (CCN), mutational analyses were conducted at key substrate recognition sites, Asp187 and Ser188, and their effects on substrate specificity and enzymatic activity were evaluated. Mutants with the Asp187 substitution exhibited a significant reduction in catalytic activity compared with the wild-type enzyme, whereas the Ser188 mutants displayed a comparatively minor effect on activity. This indicates that Asp187 plays a crucial role in catalytic function, whereas Ser188 serves a complementary role in substrate recognition. Interestingly, the substitution of the Asp187 to Glu or Ser caused novel substrate specificities, resulting in the recognition of Orn and His residues. In addition, when Asp187 and Ser188 were substituted with acidic residues (Glu or Asp), both the precursor proCCN and mature CCN proteins retained highly similar secondary and tertiary structures. This reveals that the structural characteristics of precursor proteins are maintained in the mature proteins, potentially influencing substrate recognition and catalytic function. These findings suggest that the pro-regions of these mutants interact much more tightly with the mature enzyme than in the wild-type CCN. These results provide fruitful insights into the structural determinants governing substrate recognition in enzyme variants.

## 1. Introduction

Serine proteases are a diverse group of enzymes that play crucial roles in various biological functions, including digestion, blood clotting, and immune responses. These enzymes are distinguished by their ability to catalyze the hydrolysis of peptide bonds through a mechanism involving a catalytic triad composed of Ser195, His57, and Asp102 (chymotrypsinogen numbering used throughout) [1,2]. Within the family of trypsin-like serine proteases, the amino acid residues at the base of the substrate-binding pocket, such as the Asp189 and Ser190 residues of trypsin, are particularly important due to their contributions to substrate specificity and catalytic activity [3].

The Asp189 residue, located in the S1 site (Asp189, Ser190, etc.) of trypsin [4], is a key determinant of substrate specificity. The Asp189 residue carries a negative charge, creating an electrostatic attraction with the positively charged side chains of basic residues (Lys or Arg) in the substrate [5]. This interaction is crucial for the enzyme’s preference for basic residues at the P1 position of the substrate. Mutations at the Asp189 residue, such as Asp189Ser (D189S), disrupt this interaction, leading to altered substrate specificity and reduced catalytic efficiency [6]. Furthermore, the role of the Asp189 residue extends beyond substrate binding; it also impacts the enzyme’s overall structure and its stability, underscoring its significance in maintaining enzymatic function [7].

Adjacent to the Asp189 residue, the Ser190 residue plays a complementary role in modulating substrate specificity. The Ser190 residue contributes to stabilizing the substrate within the S1 site through hydrogen bonding, facilitating the precise positioning of the substrate side chains [3,8]. The presence of Ser190 in trypsin is associated with a higher specificity for Arg than for Lys, in contrast to other serine proteases where this position may be occupied by Ala or other residues [9]. Mutations at the Ser190 can significantly affect the enzyme’s ability to recognize and cleave substrates, further elucidating its role in fine-tuning substrate specificity.

Recent studies have highlighted the intricate balance between the Asp189 and Ser190 residues in shaping the substrate-binding pocket. For instance, while the Asp189 residue primarily governs the electrostatic interaction with the substrate, the Ser190 residue enhances this interaction by stabilizing the substrate side chains through additional hydrogen bonding [4]. This dynamic interplay is essential for the catalytic function and specificity of enzymes.

Cocoonase (CCN), a trypsin-like enzyme derived from the silkworm *Bombyx mori*, is synthesized as an inactive zymogen precursor and is activated through the removal of its N-terminal propeptide [10,11]. The enzyme is known to be kinetically trapped in a molten globule-like state, transitioning to its native conformation with the assistance of the propeptide region as an intramolecular chaperone [12,13]. However, the interactions between the propeptide and mature regions of cocoonase have not yet been determined in detail, and even for trypsin, a well-studied enzyme, information on the interactions between the N-terminal propeptide and mature regions is unavailable due to the disordered structure of the propeptide. While extensive mutational analyses have been reported for trypsin and chymotrypsin, the substrate recognition mechanisms of cocoonase remain largely unexplored. Generally, mutant trypsin proteins are recombinantly obtained from the periplasmic space of *E. coli* cells and activated by enterokinase due to low yields from refolding and self-processing reactions [6]. Similarly, cocoonase is recombinantly expressed as inclusion bodies in *E. coli* cells, refolded, and self-processed for activation [12]. To eliminate non-specific degradation, a degradation-suppressed mutant of cocoonase has been successfully prepared using site-directed mutagenesis [14]. Thus, the development of degradation-suppressed cocoonase (CCN′) facilitates the investigation of both the proteolytic activities of mutant proteins and the folding mechanisms of both CCN and proCCN, thereby overcoming the challenges associated with the strong degradation activity typical of trypsin-like enzymes [14].

In this study, based on our strategy (Supplementary Figure S1), we investigated the mechanisms of substrate recognition in cocoonase, focusing specifically on the roles of the putative substrate recognition residues Asp187 and Ser188, which correspond to Asp189 and Ser190 in trypsin, respectively. The degradation-suppressed forms of the mutant precursor proteins (proCCN′ proteins) were prepared, refolded, and activated by enterokinase. This study explored the effects of mutations at the positions of Asp187 and Ser188 in cocoonase, focusing on how these mutations influence substrate recognition and catalytic activity. Our findings reveal that these mutations result in a significant reduction in catalytic activity, with altered cleavage patterns that differed from those of the wild-type cocoonase. Moreover, mutations that substitute the Asp187 and Ser188 residues with acidic residues (Glu or Asp) do not markedly change the secondary or tertiary structures of the proCCN′ and mature CCN′ proteins, indicating that the two forms maintain similar structural features. This suggests that these mutants retain the structural memory of the precursor state, referred to as “protein memory” [15].

## 2. Results

### 2.1. Molecular Modeling for Determining the Putative Substrate Recognition Site

**Figure 1:**
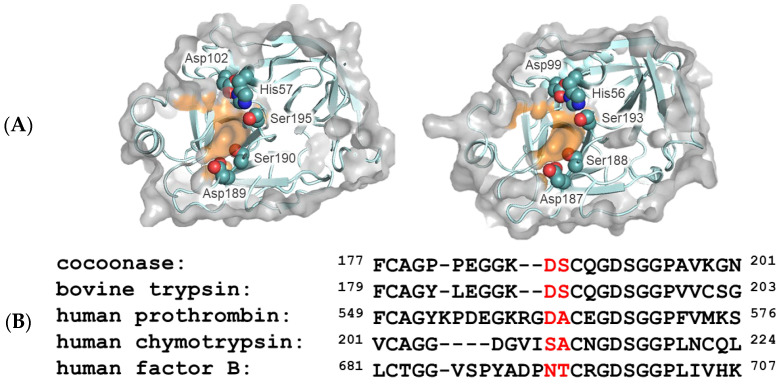
Molecular modeling of trypsin and cocoonase and their substrate-binding regions. (**A**) The molecular structures of the substrate-binding regions of trypsin (PDB: 5T3H, left) and *Bombyx mori* cocoonase (calculated using AlphaFold2, right). Surfaces of the amino acid residues at the S1 site are highlighted in orange. The catalytic triad and substrate-binding residues are indicated. (**B**) Alignment of the amino acid sequences of the substrate-binding regions of trypsin-like proteases. The predominant substrate-binding residues are indicated by red letters.

The substrate specificity of serine proteases is generally focused on the P1/S1 interactions, as described by Schechter and Berger [16]. To explore the substrate-binding residues (S1 site) of *Bombyx mori* cocoonase, the 3D modeling program AlphaFold2 was employed [17]. Compared to the primary S1 site (Asp189, Ser190, etc.) of trypsin, the putative substrate-binding residues of cocoonase were assigned to Asp187, Ser188, etc., along with those of trypsin, as shown in Figure 1. It is well established that the Asp189 and Ser190 residues of trypsin predominantly facilitate the interaction with the substrate molecule [1], as described in Section 1. Based on the structural comparison and sequence alignment of serine proteases in Figure 1A,B, the Asp187 and Ser188 residues of cocoonase were assigned as putative substrate-binding residues. Therefore, Asp187 and Ser188 in cocoonase were selected as mutation sites for elucidating the substrate recognition mechanism.

### 2.2. Preparation of Precursor and Mature Proteins of Cocoonase Mutants

The Asp189 and Ser190 residues play key and complementary roles in the substrate specificity of trypsin, respectively [3]. To investigate the role of the Asp187 residue of cocoonase in substrate specificity, we utilized the degradation-suppressed prococoonase (proCCN′) [14] and introduced a series of amino acid residues, such as Glu, Ser, Asn, and Ala residues, to the position of 187 using site-directed mutagenesis (Supplementary Table S1). The Glu and Ala residues in the mutant proteins were selected to evaluate the role of the negative charge of Asp187 in substrate recognition. In addition, Ser and Asn in chymotrypsin and factor B occupy the same relative positions of Asp189 in trypsin, respectively, as shown in Figure 1B.

The mutant proteins were successfully expressed in *E. coli* cells, refolded, and purified by cation exchange chromatography, as previously reported [12]. However, the [K8D,D187E]-proCCN′ protein was not self-processed due to significantly reduced enzymatic activity. To address this issue, we introduced an enterokinase recognition sequence (DDDDK) at the processing site to achieve the maturation of mutant proteins, as summarized in Supplementary Table S2. The cassette-[D187E]-proCCN′ protein, of which the cassette represents the [K8D,E10D,E11D] mutation, was well expressed using the *E. coli* expression system, refolded, and purified by cation exchange chromatography, as shown in Supplementary Figure S2A [12]. The purified cassette-[D187E]-proCCN′ protein was subsequently treated with enterokinase for activation, yielding the mature [D187E]-CCN′ protein, as shown in Supplementary Figure S2B. Thus, the precursor and mature forms of the [D187E]-CCN’ protein were successfully obtained. A series of precursor proteins in which the Asp187 residue was replaced with Ala, Asn, or Ser was also prepared alongside the cassette mutation. The recombinant precursor proteins were well expressed, refolded, and purified by cation exchange chromatography, as well as the cassette-[D187E]-proCCN′ protein. The mutant precursor proteins were then treated with enterokinase, resulting in the production of mature mutant proteins, such as [D187A]-, [D187N]-, and [D187S]-CCN′, as shown in Supplementary Figure S2B.

The precursor proteins of CCN′ mutant proteins in which the Ser188 residue was replaced with Asp, Glu, or Ala were prepared using the previously described method. The precursor and mature proteins were purified by cation exchange chromatography, as shown in Supplementary Figure S2C,D. Thus, the precursor and mature proteins, such as the [S188A]-, [S188D]-, [S188E]-, [D187E,S188D]-, and [D187E,S188E]-CCN′ proteins, were successfully obtained.

### 2.3. Casein Zymography and Casein Assay of Mutant Proteins

**Figure 2:**
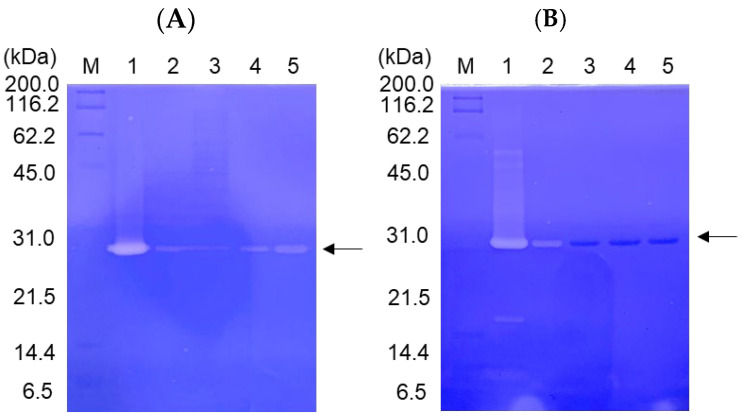
Casein zymography of the cocoonase (CCN) proteins mutated at substrate-binding sites, Asp187 (**A**) and Ser188 (**B**). (**A**) Lanes 1–5 represent the CCN′ and [D187E]-, [D187A]-, [D187N]-, and [D187S]-CCN′ proteins, respectively. (**B**) Lanes 1–5 represent the [S188A]-, [S188D]-, [S188E]-, [D187E,S188D]-, and [D187E,S188E]-CCN′ proteins, respectively. The CCN′ protein at lane 1 was derived from the [K8D]-proCCN′ protein. “M” represents marker proteins.

The enzyme activities of the CCN′ mutant proteins were qualitatively assessed using casein zymography, as shown in Figure 2. The mutation at Asp187 resulted in a drastic reduction in the enzyme activity (Figure 2A), as well as that in trypsin [3]. In contrast, the substitution of Ser188 with Ala did not significantly affect the enzyme activity, and the [S188D]-CCN′ protein still exhibited weak proteolytic activity (Figure 2B), consistent with the behavior of [S190A]- and [S190D]-trypsin mutants [3]. To evaluate the negative charge at Asp187 and Ser188, the [S188D]-, [S188E]-, [D187E,S188D]-, and [D187E,S188E]-CCN′ mutant proteins were prepared, and their proteolytic activities were examined using casein zymography, as shown in Figure 2B. Unfortunately, the proteolytic activities of the mutant proteins were not detectable in the assay, except for [S188D]-CCN′. This can be attributed more to conformational effects than to the effects of negative charges. This will be further explained in Section 3.

To further investigate the kinetic parameters of the CCN′ mutant proteins, a BAEE assay was conducted. The results are summarized in Table 1. The CCN′ protein derived from the cassette-proCCN′ protein ([K8D,E10D,E11D]-proCCN′) displayed enzymatic activity nearly equivalent to that of the original CCN′ [14], suggesting that the cassette mutation in the propeptide region is not essential for enzyme activation, as previously reported [14]. However, it was challenging to determine the *k*_cat_ and *K*_m_ values of the CCN′ mutants at Asp187 under the experimental conditions, as the catalytic activities of these mutant proteins were significantly diminished. These results indicate that the Asp187 residue of cocoonase plays a critical role in substrate recognition, as well as that of trypsin [3].

The [S188A]-CCN′ protein exhibited BAEE hydrolysis activity similar to that of the original CCN′, whereas the activity of the [S188D]-CCN′ protein was undetectable in the BAEE assay, as summarized in Table 1, indicating that the hydroxyl group of the Ser residue is not essential for substrate recognition. These results are consistent with observations in trypsin [9], indicating the complementary role of the Ser residue in substrate recognition.

**Figure 3:**
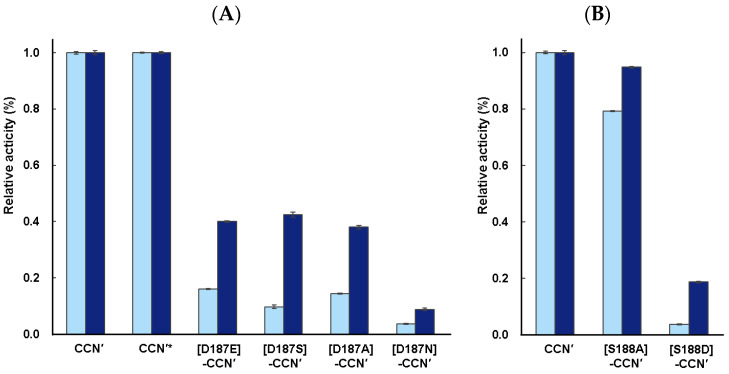
Casein assay of the CCN′ proteins mutated at putative substrate-binding sites, (**A**) Asp187 and (**B**) Ser188. The reaction times were 3 h (light blue) and 16 h (blue). Enzyme assays were conducted in triplicate (n = 3). The enzymatic activity of the CCN′ protein was normalized to the proteolytic activity of 1.0. * This CCN′ protein was derived from cassette-proCCN′.

To further assess the enzyme activities of the mutant proteins, a casein assay (casein degradation activity) was employed to evaluate the relative proteolytic activities (*k*_cat_/*K*_m_) of the mutants [18]. The casein assays were conducted as described in Section 4. The mature CCN′ protein derived from cassette-proCCN′ showed proteolytic activity nearly identical to that of the CCN′ protein derived from proCCN′, as shown in Figure 3A. This result further supports the conclusion that the cassette mutation does not affect the enzyme activity, as previously mentioned.

The protease activities of the [D187E]-, [D187S]-, [D187A]-, and [D187N]-CCN′ proteins were successfully measured, although they exhibited relatively low protease activities (*k*_cat_/*K*_m_) under the conditions used in this study. The apparent protease activity followed the order [D187E]-CCN′ ≅ [D187A]-CCN′ ≅ [D187S]-CCN′ > [D187N]-CCN′, irrespective of the reaction time (3 h or 16 h), as shown in Figure 3A. Mutational analysis in trypsin showed that trypsin mutants such as the [D189E]-, [D189S]-, [D189A]-, and [D189N]-trypsin retain weak enzymatic activities [3,6]. The [D189E]-trypsin mutant displayed higher protease activity than the other mutant proteins, although the enzyme possessed a proteolytic activity (*k*_cat_/*K*_m_) with a 3-order magnitude decrease in *k*_cat_/*K*_m_, highlighting the importance of the negative charge at position 189 (Asp189) for substrate recognition [3]. Indeed, the [D189S]-trypsin mutant protein exhibited drastically reduced activity with a 5-order magnitude decrease in *k*_cat_/*K*_m_ [6]. As described above, the mutational analysis of cocoonase proteins with substitutions at the Asp187 residue provided slightly different results for enzyme activity compared with trypsin. The [D187E]-CCN′ protein showed proteolytic activity similar to those of the [D187A]- and [D187S]-CCN′ proteins. The observed differences in activity may be attributed to variations in the substrates used. Specifically, when casein was employed, the mutants could recognize and hydrolyze multiple sites, and the measured activity reflected a cumulative effect. To validate this possibility, the substrate specificity of these mutants was examined.

Additionally, mutant proteins in which the Ser188 residue was replaced by either Ala or Asp were prepared to further investigate substrate recognition in cocoonase. As expected, the [S188A]-CCN′ protein exhibited strong proteolytic activity in the assay, whereas the activity of the [S188D]-CCN′ protein was significantly weaker, as shown in Figure 3B. These results are consistent with those of trypsin mutants [9], indicating that the Ser residue at the S1 site plays a complementary role in substrate recognition of cocoonase.

### 2.4. Substrate Specificity of Mutant Proteins

**Figure 4:**
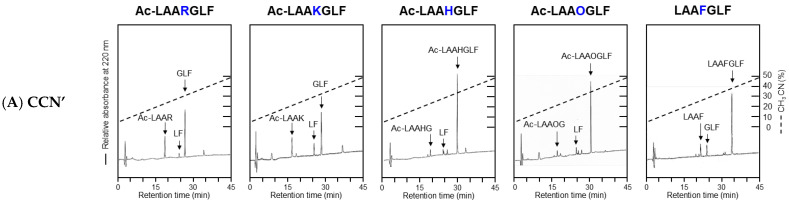

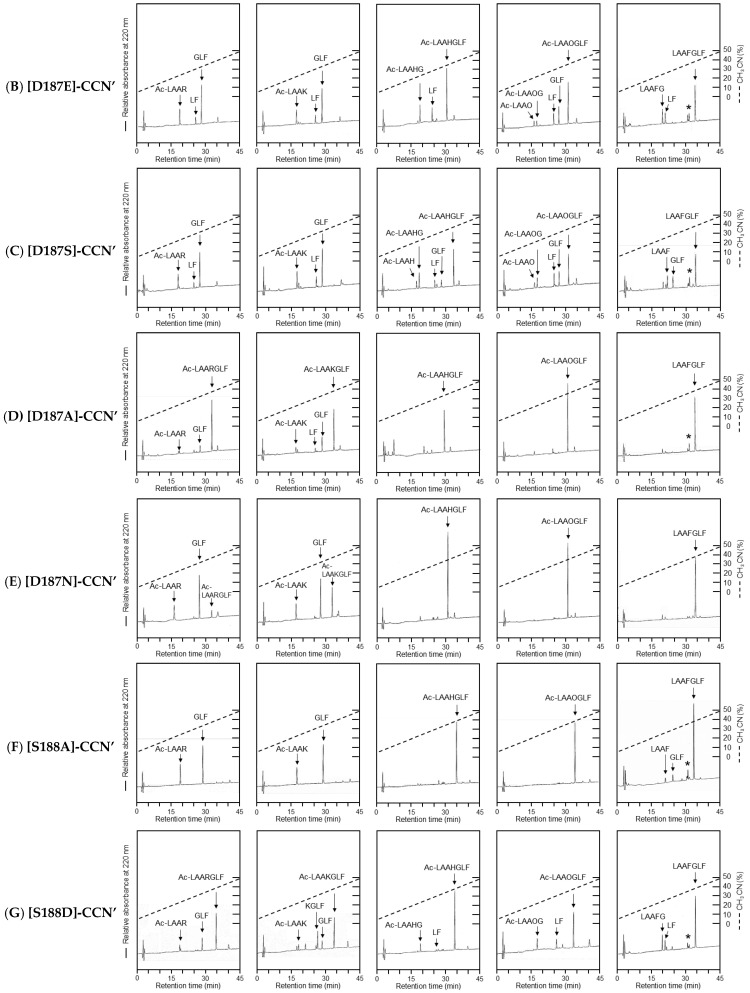

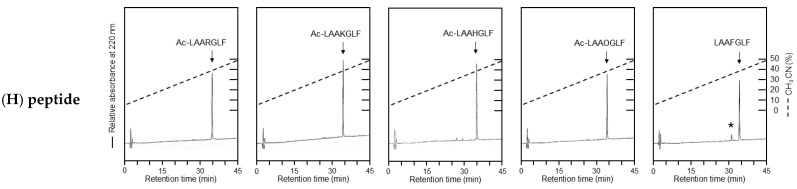
HPLC profiles of the peptide substrates treated with the CCN′ mutant proteins. The peptide fragments were analyzed by amino acid analysis. Their identified sequences are indicated in the profiles. (**A**) The CCN′ protein was derived from [K8D]-proCCN′. (**B**–**G**) represent [D187E]-, [D187S]-, [D187A]-, [D187N]-, [S188A]-, and [S188D]-CCN′. (**H**) The peptide substrates were allowed to stand for 3 days at 37 °C without the mutant proteins. There were no significant digests in the absence of the mutant enzymes. Asterisks (*) indicate impurities derived from the LAAFGLF peptide.

**Figure 5:**
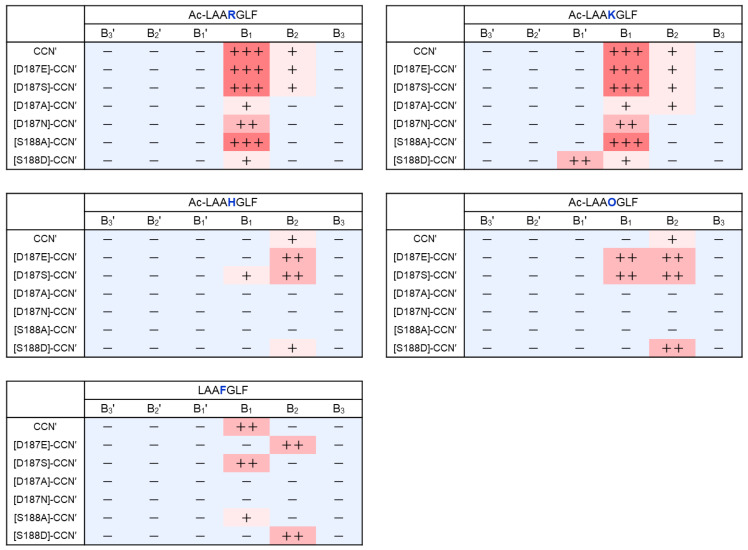
Heat map of substrate specificity of the CCN′ mutant proteins. The cleaved sites identified by the mutant proteins are summarized, with the relative strength of cleavage represented using the number of “+” symbols. +++: strong, ++: moderate, +: weak, −: not detected.

The substrate specificity of the cocoonase mutant proteins was evaluated using a series of synthetic peptides. For this purpose, a set of peptides (Ac)-LAAXGLF was designed, where X represents the specific residue recognized by cocoonase, allowing for cleavage sites to be positioned in the central regions of the peptides, as shown in Supplementary Figure S3. To evaluate the substrate specificity of the mutant enzymes, Arg, Lys, His, Orn, and Phe were employed at the position of “X” in the peptide substrates. Enzyme digestion was conducted for 3 days under the conditions described in Section 4, as the proteolytic activities of the mutant proteins were significantly low. This is also a merit to using the degradation-suppressed cocoonase because the self-digestion of the CCN′ mutant proteins and non-specific digests of the substrates were not significant. Subsequently, the enzyme digests were subjected to RP-HPLC, as shown in Figure 4. The peptide bonds cleaved by the mutant proteins were summarized, with the strength of cleavage represented using the number of “+” symbols, as depicted in Figure 5.

The CCN′ protein cleaved the B_1_ sites of the Arg and Lys residues in Ac-LAARGLF and Ac-LAAKGLF, respectively, producing the anticipated peptide fragments, as shown in Figure 4A. In addition, the enzyme recognized and cleaved the B_1_ site in LAAFGLF, indicating that cocoonase has the chymotrypsin activity. This additional ability was also observed in trypsin [5]. Surprisingly, the enzyme also showed proteolytic activity at the B_2_ sites of these peptides, resulting in the generation of the Leu-Phe (LF) peptide. It seems improbable that this enzyme recognizes Gly; therefore, cleavage likely occurred at the N-terminal peptide bond of the Leu residue. Although the activity appeared to be chymotrypsin-like, cleavage at the B_2_ site was not observed when LAAFGLF, which contains Phe as a substrate, was used.

The [D187E]-CCN′ protein was still able to recognize the Arg and Lys residues, cleaving at the B_1_ site along with proteolytic activity at the B_2_ site, similar to that of the CCN′ protein, as shown in Figure 4B. Notably, the mutant protein cleaved the B_1_ site of the Orn residue in Ac-LAAOGLF, as shown in Figure 4B. This result indicates that the extension of one methylene group at the side chain of the Asp187 residue caused substrate specificity for Orn, of which the side chain was shorter than that of Lys by just one methylene group. Interestingly, the chymotrypsin-like activity of the enzyme at the B_1_ site was lost, as shown at LAAFGLF in Figure 4B.

The [D187S]-CCN′ protein exhibited similar results to those of the CCN′ protein, as shown in Figure 4C. Importantly, the mutation induced a novel catalytic activity at the His residue in Ac-LAAHGLF, as shown in Figure 4C. This enzyme activity was observed only for the [D187S]-CCN′ mutant protein among the mutant proteins prepared in this study. In addition, the mutant protein exhibited slightly higher proteolytic ability at the B_1_ site of the Phe residue compared with the CCN′ protein. This indicates that the substitution of the Asp187 residue with Ser induces chymotrypsin-like ability in cocoonase, as well as that observed in trypsin [5]. It was reported that the Ser residue in the [D189S]-trypsin mutant protein, which is located at a position analogous to D187S in cocoonase, interacted with the substrate via an acetate ion in the crystal structure [6]. Interestingly, the reduced activity of the [D189S]-trypsin mutant was restored by the addition of exogenous acetate, highlighting the critical role of the negative charge at the base of trypsin’s binding pocket [19]. Therefore, to investigate the role of negative charge at the substrate recognition site of cocoonase, the peptide substrate (Ac-LAARGLF) was treated with the [D187S]-CCN′ mutant protein in the presence of 2 M sodium acetate. The proteolytic activity of the [D187S]-CCN′ mutant protein was significantly increased in the presence of acetate, resulting in greater amounts of proteolytic products under these conditions (Supplementary Figure S4). Thus, acetate ions provide the necessary negative charge at the S1 site in the binding pocket and facilitate substrate binding of cocoonase.

The [D187A]-CCN′ protein exhibited weak protease activity against the peptide substrates, as shown in Figure 4D, although its proteolytic activity against casein was similar to those of the [D187E]- and [D187S]-CCN′ proteins. This observation reflects a disparity in affinity between peptide and protein substrates. The *K*_m_ values for peptide substrates may be significantly larger than those for the protein substrate, such as casein. In contrast, the [D187N]-CCN′ protein exhibited slightly stronger protease activity for Ac-LAARGLF and Ac-LAAKLGF than the [D187A]-CCN′ protein, as shown in Figure 4D,E. The [D187N]- and [D187A]-CCN′ proteins provided no cleaved products for the peptide substrates, including Ac-LAAHGLF, Ac-LAAOGLF, and LAAFGLF.

The Ala substitution at Ser188 did not significantly affect the enzyme activity (*k*_cat_/*K*_m_) or substrate specificity, as shown in Figure 3B and Figure 4F. However, the Leu-Phe peptide was not observed in the digestion of the peptide substrates by the [S188A]-CCN′ protein, implying that the Ser residue is involved in the hydrolysis of the N-terminal peptide bond of the recognized amino acid residue through hydrogen bonding. Thus, the mutant enzyme showed distinct substrate specificity against Arg and Lys compared with the original cocoonase.

Notably, the [S188D]-CCN′ protein cleaved the B_1_ site and, interestingly, also cleaved the B_1_′ site of the Lys residue in Ac-LAAKGLF, although its proteolytic activity was slightly lower, as shown in Figure 4G. These results suggest that the Ser188 residue contributes to the hydrolysis mechanism not only by forming hydrogen bonds with the substrate (including water molecules) but also by aiding in the formation of oxyanion hole [20].

### 2.5. Structural Analysis of the proCCN′ and CCN′ Mutant Proteins

**Figure 6:**
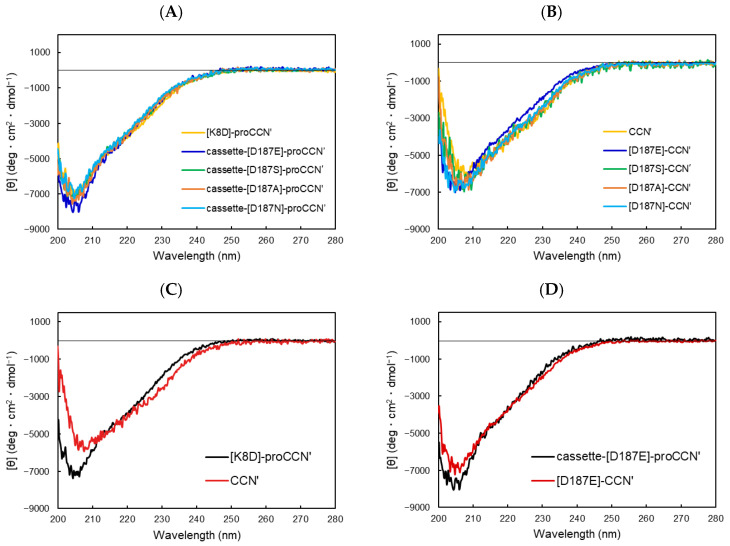
CD spectra of a series of the proCCN′ (**A**) and CCN′ (**B**) mutant proteins. (**A**) Precursor proteins, (**B**) mature proteins, (**C**) [K8D]-proCCN′ and CCN′, and (**D**) cassette-[D187E]-proCCN′ and [D187E]-CCN′.

To obtain structural information about the mutant proteins, circular dichroism (CD) measurements were performed, as shown in Figure 6. The CD spectra of all proCCN′ mutant proteins overlapped well (Figure 6A), indicating that the mutant proteins were folded correctly. The mature CCN′ proteins also exhibited similar spectra, except for the [D187E]-CCN′ protein, as shown in Figure 6B. It has been reported that the precursor form of cocoonase (proCCN′) displays a slightly different spectrum from that of the mature protein CCN′ (Figure 6C). Interestingly, the CD spectrum of the [D187E]-CCN′ protein resembled that of its precursor protein, [D187E]-proCCN′, as shown in Figure 6D. This indicates that the conformation of the precursor protein was retained in the mature enzyme, suggesting that the mutation at the substrate-binding site (Asp187) induced a phenomenon known as “protein memory” [15]. This phenomenon was also observed in the CD spectra of the [S188D]-proCCN′ and [S188D]-CCN′ proteins, as depicted in Supplementary Figure S5. These results suggest that the Glu187 residue and the exogenous negative charge at Ser188 trapped the local conformation required for the dynamic movement from the precursor to mature proteins.

## 3. Discussion

This study focused on elucidating the effects of mutations at the putative substrate recognition sites Asp187 and Ser188 in cocoonase, a trypsin-like serine protease. By altering these putative substrate recognition residues, we provide novel and valuable insights into how such modifications influence substrate recognition, marking the first detailed investigation of the substrate recognition mechanism of cocoonase by the site-directed mutagenesis.

All mutant proteins still retained their catalytic activity against peptide substrates containing Arg or Lys, while the proteolytic activity of mutant proteins, except for S188A, was markedly reduced compared with that of the wild-type cocoonase. These results reveal that Asp187 and Ser188 in cocoonase are involved in the recognition of the substrates Arg and Lys, as well as that in trypsin. However, certain mutant proteins acquired the additional ability to recognize different sequences (Figure 5). Remarkably, the replacement of Asp187 with Glu or Ser enabled it to recognize different substrates, such as His and Orn. In addition, the [S188D]-CCN′ protein cleaved the B_1_′ site of Ac-LAAKGLF, which was entirely unexpected. These results indicate that Asp187 and Ser188 in cocoonase are involved in the recognition of the substrate residues Arg and Lys, as well as that in trypsin.

To further investigate the decreased enzymatic activity observed in the mutants, we employed CASTp to calculate the volume of the substrate-binding pocket, as summarized in Supplementary Table S3. Mutants with a similar volume for the binding pocket to that of wild-type CCN or [K8D]-CCN′ (33–38 Å^3^) still retained sufficient activity, as shown in Supplementary Figure S6. However, when the pocket volume exceeded 38 Å^3^, a sharp decline in activity was noted, likely due to reduced substrate affinity caused by the expanded pocket, as shown in Supplementary Figure S6. Conversely, when the volume was smaller than 33 Å^3^, the activity dropped significantly, suggesting that the substrate could not properly access the binding site. Interestingly, the [S188D]-CCN′ mutant deviated from this trend, possibly because the hydroxyl group of the residue facilitated direct hydrogen bonding with Arg and Lys, as that observed in a trypsin mutant [3]. These results also reflect findings in thrombin and further emphasize the importance of specific residues in cocoonase’s substrate recognition [5].

Surprisingly, our data support the notion of “protein memory”, in which the secondary and tertiary structures of the precursor proCCN are preserved in the mature enzyme [15]. In this regard, we hypothesized that the propeptide region of cocoonase interacts with the substrate-binding residue(s) to form a distinct conformation of the substrate-binding pocket and that the removal of the propeptide region induces a dynamic conformational change in the enzyme molecule for its activation. In fact, the dynamic movement of N-terminal Ile16 induces enzyme activation through its ionic interaction with Asp194 [1,21]. Substitution of the Asp187 with Glu extensively reduces the proteolytic activity of cocoonase in the BAEE assay. While a decrease in activity due to such substitutions has also been reported in the case of trypsin [3], the impact appears to be more significant in cocoonase. These observations may be attributed to the influence of protein memory in cocoonase, which allows for the retention of the precursor structure. Consequently, the structural alterations required for enzyme activation, particularly in the N-terminal region, may be inadequate, resulting in diminished enzymatic activity. This result also implies that the interaction between the N-terminal propeptide and mature region is critical for substrate recognition and catalytic activity. The absence of dynamic structural changes following propeptide cleavage in these mutants could lead to reduced stabilization of the oxyanion hole, thereby lowering the enzyme efficiency. Our findings suggest that these conformational changes are diminished in cocoonase mutants, possibly contributing to the reduction in catalytic performance.

We investigated the role of Asp187 in cocoonase and its interactions with adjacent residues, particularly in relation to substrate binding and structural stability. The main chain of Asp187 engages in hydrogen bonding with the carbonyl oxygen (CO) and amide nitrogen (NH) of the second residue (Val14) from the N-terminus of the mature enzyme, with bond distances of 2.8 Å and 3.1 Å, respectively (Supplementary Figure S7A). Notably, analogous interactions were observed in the crystal structure of trypsin, indicating a conserved mechanism across these enzymes. Importantly, the substitution of Asp187 with Glu does not significantly compromise these hydrogen bonds; rather, their integrity is preserved (Supplementary Figure S7B). Additionally, the side chain of Asp187 forms a hydrogen bond with the main chain of Ala214 at a distance of 3.1 Å. This bond distance decreased to 2.1 Å upon the replacement of Asp187 with Glu, resulting in a markedly enhanced interaction.

The Ala214 residue, located in the activation domain [2], engages in hydrophobic interactions with the methyl groups of the Val14 side chain in the D187E mutant protein, as shown in Supplementary Figure S7. These hydrophobic interactions, in conjunction with the previously described hydrogen bonds involving Val14 and the hydrogen bond between Glu187 and Ala214, appear to stabilize the interactions between the N-terminal region, the substrate recognition site, and the activation domain via Glu187. The significance of mutations in the substrate recognition site and their structural impact on the activation domain have been elucidated in the crystal structure of the D189S mutant protein of trypsin [22]. Structural rearrangements within the activation domain are critical for the activation of trypsin-like enzymes, as reported in multiple studies [1,21,23]. Based on these findings, we propose that the substitution of Asp187 with Glu in cocoonase enhances the interaction between the activation domain (via Ala214) and the N-terminal region (via Val14) in both the precursor and mature enzyme forms. This substitution may preserve precursor-like structural features within the mature enzyme, potentially influencing the integrity of the ionic bridge between Ile13 and Asp192 in cocoonase, which is assumed to be essential for enzymatic activation. Consequently, the D187E mutant is hypothesized to exhibit reduced enzymatic activity compared to a fully active enzyme. Additionally, this enhanced interaction could serve as a driving force for “protein memory”, impeding structural transitions from the precursor to the mature form. Therefore, we hypothesize that the mutation from Asp187 to Glu promotes robust interactions with Ala214, trapping the structural memory of the precursor protein. This interaction likely mediates structural regulation at the substrate-binding site, ultimately hindering the transition from the precursor state to the mature state.

## 4. Materials and Methods

Boc-amino acids, HBTU, HOBt, glutathione, and Bz-Arg-OEt (BAEE) were purchased from the Peptide Institute, Inc. (Osaka, Japan). Casein was purchased from FujiFilm Wako Pure Chemicals, Ltd. (Osaka, Japan). All chemicals and solvents used were of reagent grade. Protein markers in SDS-PAGE were purchased from Nacalai tesque (Kyoto, Japan).

### 4.1. Construction of Expression Vectors of the Recombinant Mutant Proteins for an E. coli Expression System

The cDNAs encoding the amino acid sequences of the mutant proteins were prepared by PCR, as previously reported [12]. The primer sequences used in this study are summarized in Supplementary Table S1. First, the cDNA of the cassette mutant protein, in which the propeptide sequence (KDEEK) was replaced with the enterokinase recognition sequence (DDDDK), was prepared by PCR using the cDNA of the degradation-suppressed [K8D,K63G,K131G,K133A]-proCCN protein as a template [14]. The PCR reaction was performed using Platinum *Pfx* DNA polymerase (Invitrogen; Thermo Fisher Scientific, Inc., Carlsbad, CA, USA), as previously described [12]. Then, the amplified cDNA’s were subcloned into the pET-17b expression vector (Novagen, Glendale, CA, USA), following the introduction of *Nde*I and *Eco*RI sites at their 5′ and 3′ ends, respectively. The resulting expression vector, referred to as pNS9, contained the cDNA of [K8D,E10D,E11D,K63G,K131G,K133A]-proCCN. The cDNA sequences of the vectors were confirmed by the DNA sequencing service of Eurofins Japan (Tokyo, Japan).

Next, other expression vectors of the proCCN′ mutant proteins were prepared by the two-step PCR method, as previously described [14], using pNS9 as a template. The resulting expression vectors are summarized in Supplementary Table S2.

### 4.2. Protein Expression and Purification of Recombinant Proteins

Protein expression was performed using a previously reported method [12]. All mutant proteins were obtained as inclusion bodies in *E. coli* cells and possessed a Met residue at the N-terminus of the proCCN′ mutant proteins, which was derived from the *Nde*I site during subcloning. After sonication of the cells, the mixtures were centrifuged at 20,000× *g* for 15 min. The residues were subsequently washed with 0.5% Triton X-100 (Nacalai tesque, Kyoto, Japan) and 50 mM sodium phosphate buffer (pH 7.0). The final products were resuspended in MilliQ water, and the protein amounts were estimated by SDS-PAGE analyses using BSA as a standard protein. The resulting mutant proCCN′ proteins were refolded by the method previously reported [24,25] and purified by cation exchange chromatography, as described below.

### 4.3. Activation of Recombinant Cocoonase by Enterokinase Treatment

The fractions of the proCCN′ mutant proteins obtained by cation exchange chromatography were concentrated to approximately 1 mg/mL protein solutions using Amicon Ultra-15 (10K, Merck Millipore, Darmstadt, Germany) at 4 °C. The proCCN′ mutant proteins (0.1 mg protein) were treated with enterokinase solution (1 mg/mL, 15 μL) at 25 °C overnight. To remove enterokinase, the reaction mixtures were further treated with EKapture^TM^ Agarose (Novagen, Glendale, CA, USA, 50 μL) pre-equilibrated with 0.2 M Tris/HCl (pH 7.4), 20 mM CaCl_2_, and 0.5 M NaCl and centrifuged at 9000× *g* for 1 min at 4 °C. The supernatants were analyzed by SDS-PAGE. Aliquots of the protein solutions were lyophilized and stored at 4 °C until use.

### 4.4. Preparation of Peptide Substrates

The peptide substrates were chemically synthesized on a Merrifield’s resin using HBTU/HOBt as a condensation reagent by the Boc solid-phase method [26]. After treatment with anhydrous hydrogen fluoride, the target peptides were purified by RP-HPLC, as described below, and were characterized by MALDI-TOF/MS and amino acid analyses [27,28].

### 4.5. Casein Assay

The enzyme activity of each CCN′ mutant protein, purified by cation exchange chromatography, was examined using casein as a substrate [29]. The mutant protein (10 µL) was mixed with either 2% casein or 50 mM Tris/HCl (pH 8.0) (190 µL) and incubated for either 3 h or 16 h. After incubation, trichloroacetic acid was added to achieve a final concentration of 2%, and the mixtures were allowed to stand for 20 min. The reaction solutions were centrifuged (20,000× *g*, 15 min, 4 °C) to separate the supernatant and pellet fractions. The resulting supernatant fractions were measured using a UV spectrometer (Eppendorf BioSpectrometer basic, Eppendorf, Hamburg, Germany). Enzyme assays were conducted in triplicate.

### 4.6. Enzyme Digestion Using Synthetic Peptides

The purified synthetic peptides (10 nmol) in Tris/HCl buffer (45 µL, pH 8.0) were treated with the CCN′ mutant proteins (0.1 mg/mL) in 20 mM sodium phosphate buffer (5 μL, pH 7.0) containing 0.3 M NaCl at 37 °C for 3 days. The reaction mixtures were then subjected to RP-HPLC.

### 4.7. Cation Exchange Chromatography

Cation exchange chromatography was performed using the ÄKTA purifier (GE Healthcare Japan, Tokyo, Japan) to purify the proCCN′ and CCN′ mutant proteins, as previously reported [12]. Briefly, the proteins were dialyzed against 50 mM phosphate buffer (pH 7.0) containing 20 mM NaCl. After dialysis, the enzyme solution was applied to a HiTrap SP HP column (1 mL, GE healthcare Japan, Tokyo, Japan) pre-equilibrated with Buffer A (50 mM phosphate buffer, pH 7.0) and eluted with Buffer B (50 mM phosphate buffer containing 1 M NaCl, pH 7.0). The eluted protein concentrations were determined by the Bradford’s method or UV absorbance.

### 4.8. Reversed-Phase High Performance Liquid Chromatography (RP-HPLC)

A Waters M600 multisolvent delivery system (Bedford, MA, USA) and a HITACHI ELITE LaChrom system L2130 (Tokyo, Japan) equipped with a Hitachi L-3000 detector and a D-2500 chromato-integrator were used for the separation of peptides and proteins, respectively. Peptides were separated by RP-HPLC using a Hydrosphere C_18_ column (4.6 × 150 mm, YMC Co., Kyoto, Japan) [30]. The peptides and proteins were eluted with a linear gradient of CH_3_CN in 0.05% TFA/H_2_O at a flow rate of 1.0 mL/min at 25 °C. The absorbance of eluates was monitored at 220 nm. The separated peptides were analyzed by amino acid and mass spectrometric analyses [26,27].

### 4.9. Circular Dichroism Measurements

The proteins purified by cation exchange chromatography were dialyzed against 20 mM sodium phosphate buffer (pH 7.0) containing 20 mM NaCl, and their concentrations were determined by UV absorbance. The CD spectra of the mutant proteins (0.1 mg/mL) were recorded on a JASCO J720 spectrometer (JASCO Corporation, Tokyo, Japan) at 25 °C.

### 4.10. Molecular Modeling of CCN′ Mutant Proteins

To obtain the coordinates of the mutant proteins, protein modeling was performed using the program AlphaFold2 software (ColabFold ver. 1.5.5) [17]. Then, the areas and volumes of substrate-binding regions were calculated using the CASTp program (ver. 3.0) [31]. The final molecular structures were depicted using the programs CASTp or Pymol (ver. 3.0.3).

## 5. Conclusions

Mutational analysis at putative substrate recognition residues revealed that the substrate recognition site of cocoonase is located at Asp187, situated at the base of the substrate pocket, similar to other trypsin-like serine proteases, with the adjacent Ser188 playing a complementary role. The results suggest that the volume of the substrate-binding pocket is crucial for substrate recognition. Mutations to acidic residues at the substrate-binding sites, such as D187E and S188D, demonstrate that the precursor protein’s structure is retained in the mature form, a phenomenon referred to as “protein memory”. This preservation could disrupt the structural transition required for enzyme activation. Thus, in addition to their role in substrate binding, the substrate recognition residues (Asp187 and Ser188) also play a crucial role in regulating enzyme activation. Structural analysis of these mutants is expected to provide important insights into the activation mechanism of cocoonase and, by extension, trypsin-like enzymes.

## Figures and Tables

**Table 1:** Kinetic parameters of BAEE hydrolysis by the CCN′ mutant proteins.

	*K*_m_ (mol/L)	*V*_max_ (mol/L/s)
Native CCN	2.03 × 10^−3^	4.17 × 10^−4^
Wild-type CCN	6.35 × 10^−4^	4.45 × 10^−4^
[K63G,K131G,K133A]-CCN *	3.72 × 10^−4^	1.43 × 10^−4^
[K8D]-CCN’	9.17 × 10^−4^	4.59 × 10^−4^
[S188A]-CCN’	1.11 × 10^−3^	2.77 × 10^−4^

* [K63G,K131G,K133A]-CCN corresponds to CCN′.

## Data Availability

Data are contained within the article and Supplementary Materials.

## Supplementary Materials

The following supporting information can be downloaded at: https://www.mdpi.com/article/10.3390/molecules29225476/s1, Figure S1: Strategy for evaluating the proteolytic activity of the CCN proteins, mutated at the substrate recognition sites; Figure S2: SDS-PAGE of the purified proCCN’ (A,C) and CCN’ (B,D) mutant proteins in which the Asp187 (A,B) or Ser188 (C,D) residues were replaced by a series of amino acid residues; Figure S3: Amino acid sequences of a series of peptide substrates; Figure S4: HPLC profiles of the reaction solutions of Ac-LAARGLF treated with the [D187S]-CCN’ protein in the absence (left) and presence (right) of 2 M sodium acetate; Figure S5: CD spectra of the cassette-[S188D]-proCCN’ and [S188D]-CCN’ proteins; Figure S6: Molecular modeling of the CCN’ mutants using the combination of CASTp and Pymol; Figure S7: Schematic representation of the hydrogen bond network involving Asp187 (A) and Glu187 (B) in cocoonase; Table S1: Primer sequences used for PCR; Table S2: Expression vectors and abbreviations for mutant proteins; Table S3: Calculated volumes of the substrate-binding pocket of CCN’ mutant proteins; Wild-type CCN.pdb, CCN′.pdb, [D187E]-CCN′.pdb, [D187A]-CCN′.pdb, [D187N]-CCN′.pdb, [D187S]-CCN′.pdb, [S188A]-CCN′.pdb, [S188D]-CCN′.pdb, [S188E]-CCN′.pdb, [D187E,S188D]-CCN′.pdb, and [D187E,S188E]-CCN′.pdb represent the coordinate data of wild-type CCN, CCN′, [D187E]-, [D187A]-, [D187N]-, [D187S]-, [S188A]-, [S188D]-, [S188E]-, [D187E,S188D]-, and [D187E,S188E]-CCN′ proteins, respectively.

## Abbreviations

BAEE: benzoyl-arginine ethyl ester; Boc, *t*-butyloxycarbonyl; CCN, wild-type cocoonase; CCN′, [K63G,K131G,K133A]-CCN; CD, circular dichroism; HBTU, hexafluorophosphate benzotriazole tetramethyl uranium; HOBt, 1-hydroxybenzotriazole; MALDI, matrix assisted laser desorption/ionization; Orn, ornithine (O as a one-letter code); RP-HPLC, reversed-phase high performance liquid chromatography; TFA, trifluoroacetic acid; TOF/MS, time of flight mass spectrometry.
