# Supplementary material for: Mutational Analysis of Substrate Recognition in Trypsin-like Protease Cocoonase: Protein Memory Induced by Alterations in Substrate-Binding Site
Source: Molecules. 2024 Nov 20;29(22):5476. doi: 10.3390/molecules29225476 (PMC11597625; doi:10.3390/molecules29225476)
Supplement: Supplementary file 1 [file molecules-29-05476-s001.zip › 2024_Molecules-SI_fin.pdf]

Supplementary Materials

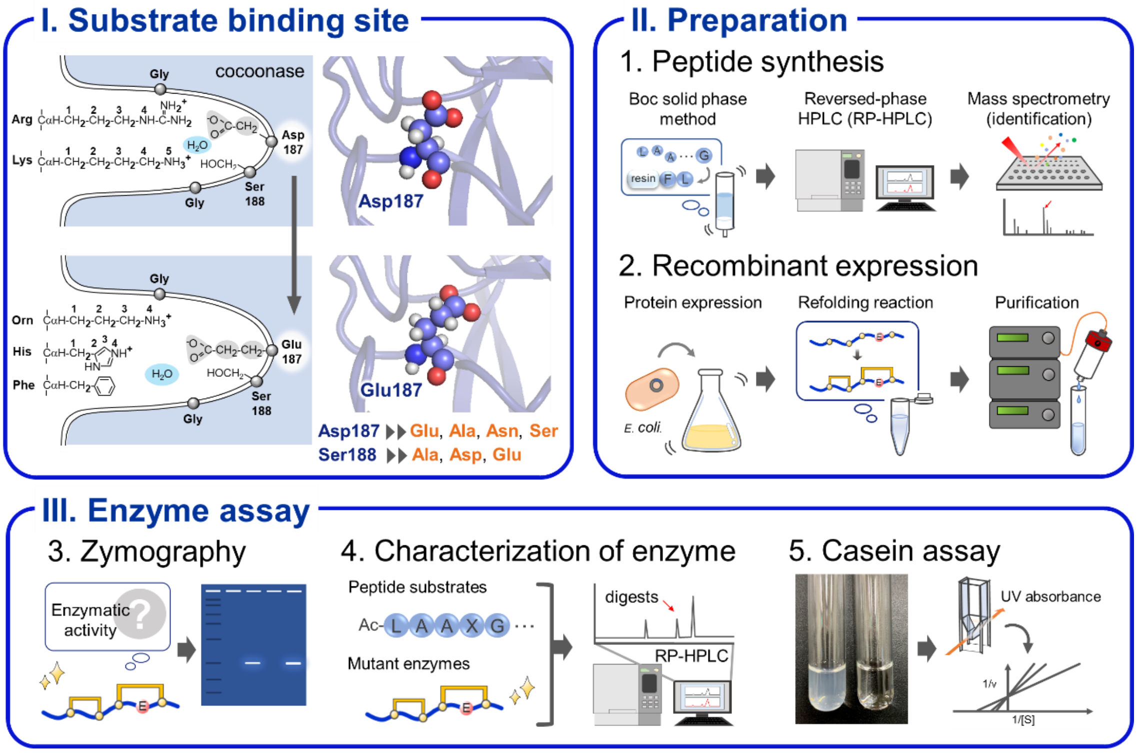

Figure S1. Strategy for evaluating the proteolytic activity of the CCN proteins, mutated at the substrate recognition sites.

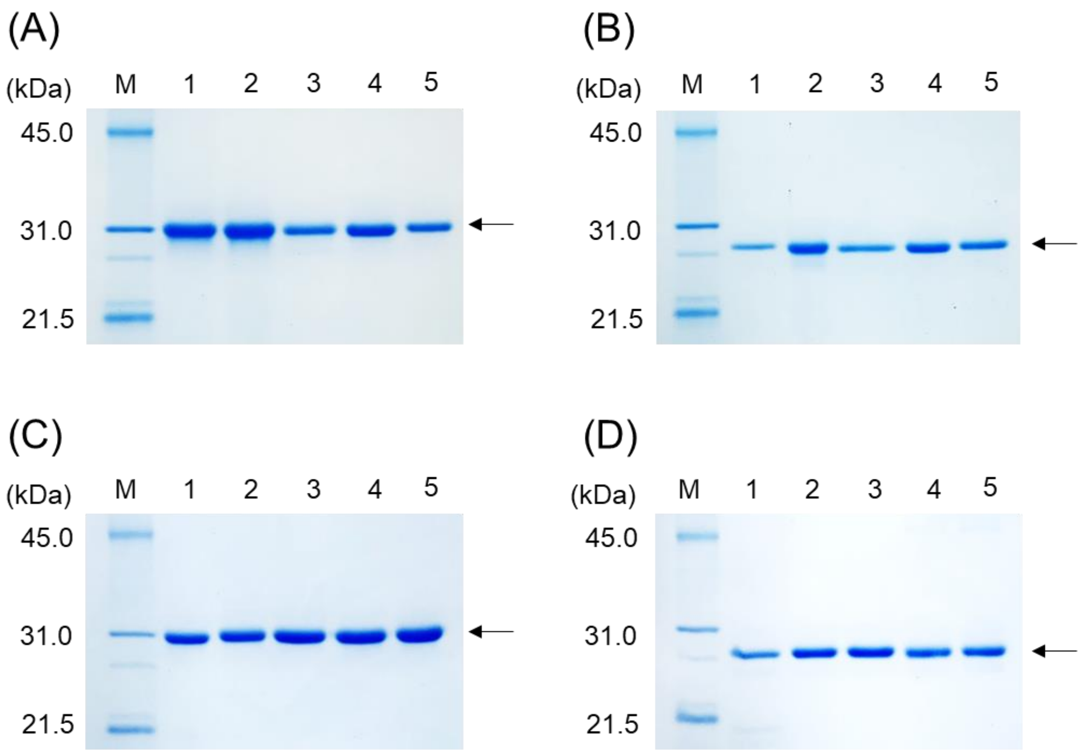

Figure S2. SDS-PAGE of the purified proCCN′ (A, C) and CCN′ (B, D) mutant proteins in which the Asp187 (A, B) or Ser188 (C, D) residues were replaced by a series of amino acid residues. The arrows indicate target proteins. A) and B): lanes 1, 2, 3, 4, and 5 represent the precursor and mature proteins of [K8D]-, [D187E]-, [D187A]-, [D187N]-, and [D187S]-CCN′ proteins, respectively. C) and D): lanes 1, 2, 3, 4, and 5 represent the precursor and mature proteins of [S188A]-, [S188D]-, [S188E]-, [D187E,S188D]-, and [D187E,S188E]-CCN′ proteins, respectively. "M" represents marker proteins.

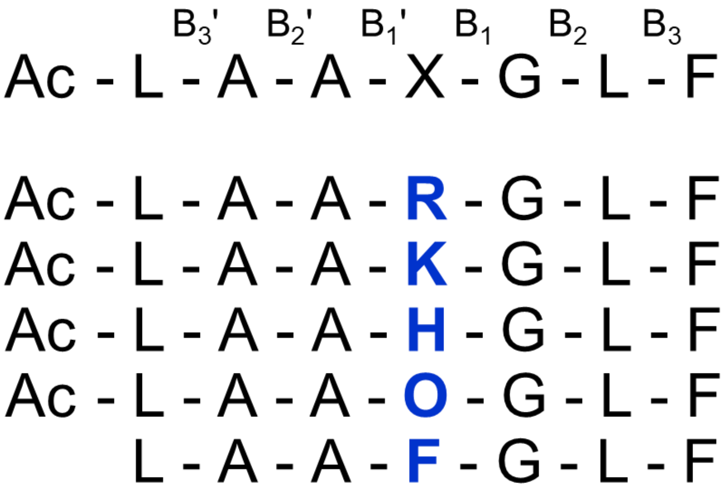

Figure S3. Amino acid sequences of a series of peptide substrates. Each peptide bond is denoted as B_1, B_2, B_3, B_1', B_2', and B_3'. The letter "X" represents the specific residue recognized by cocoonase. The letter "O" in the peptide substrate represents ornithine.

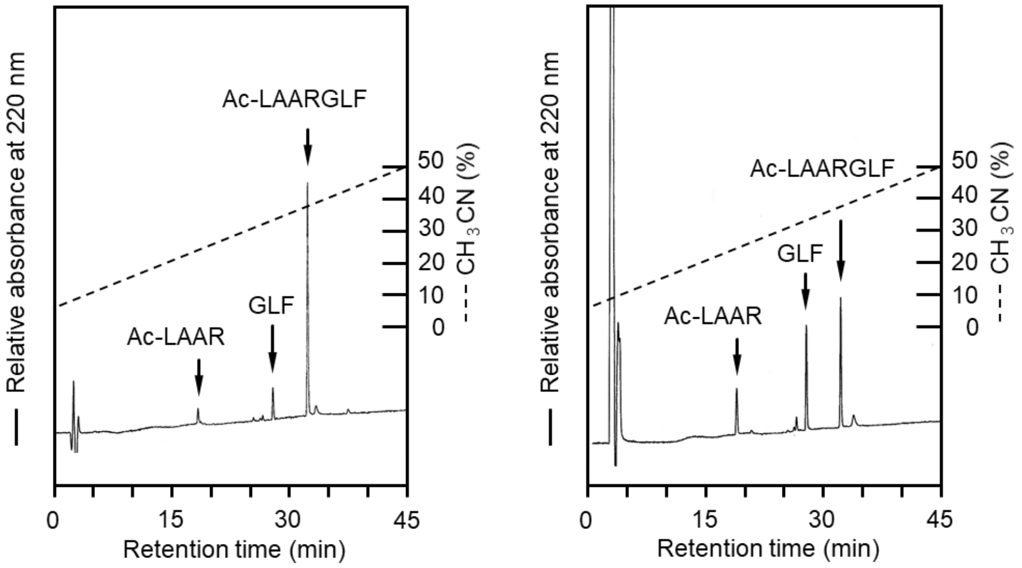

Figure S4. HPLC profiles of the reaction solutions of Ac-LAARGLF treated with the [D187S]-CCN′ protein in the absence (left) and presence (right) of 2 M sodium acetate. The peptide substrate was treated with the enzyme at 37 °C for 16 h in 50 mM Tris/HCl buffer (pH 8.0).

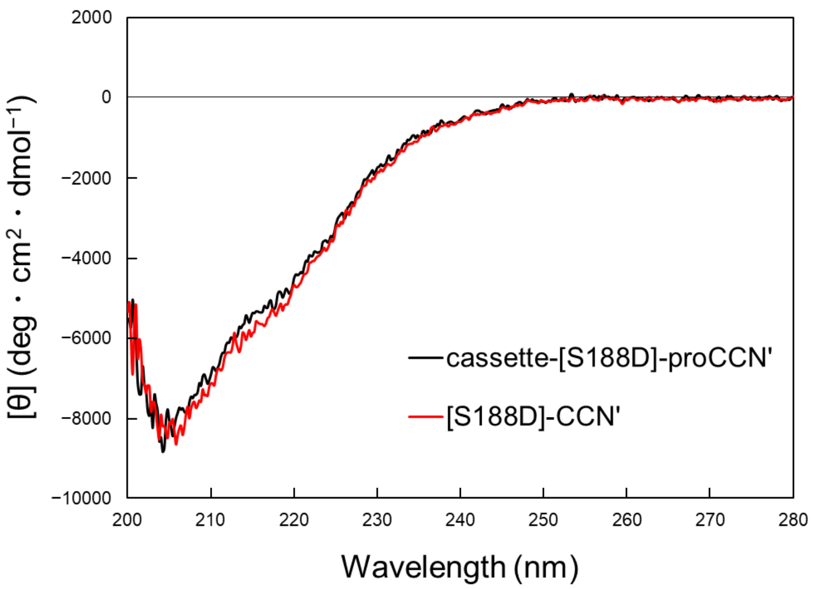

Figure S5. CD spectra of the cassette-[S188D]-proCCN′ and [S188D]-CCN′ proteins.

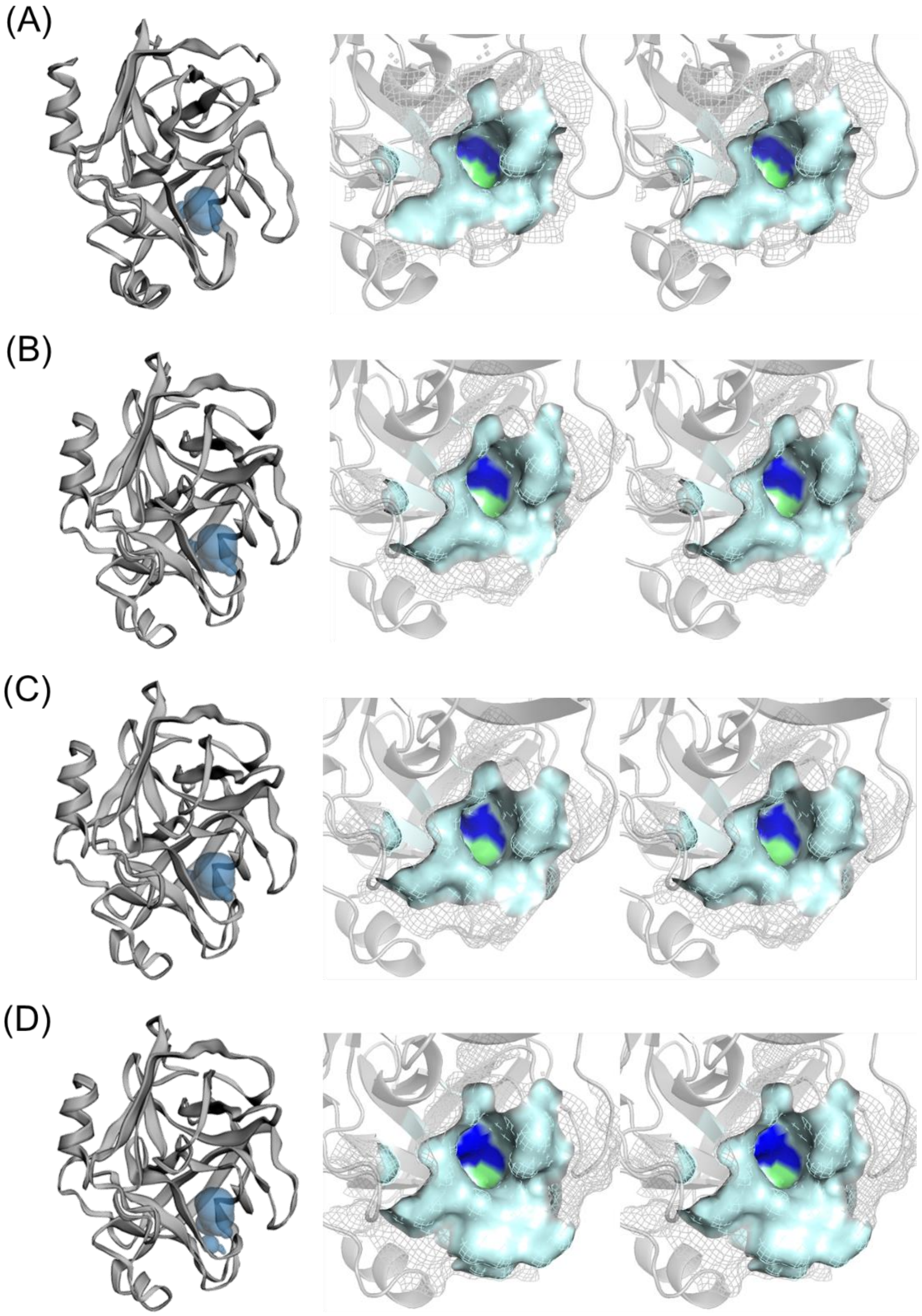

Figure S6. Molecular modeling of the CCN′ mutants using the combination of CASTp and Pymol. A) trypsin (5T3H), B) CCN, C) [D187E]-CCN′, D) [S188D]-CCN′. The calculated negative volumes are shown in blue (left). The stereo-drawings of the surfaces of the amino acid residues at positions of 187 and 188 are indicated in the center and right. The S**1** site is highlighted in green and blue. Other amino acid residues at the S1 site are highlighted in light blue (center and right).

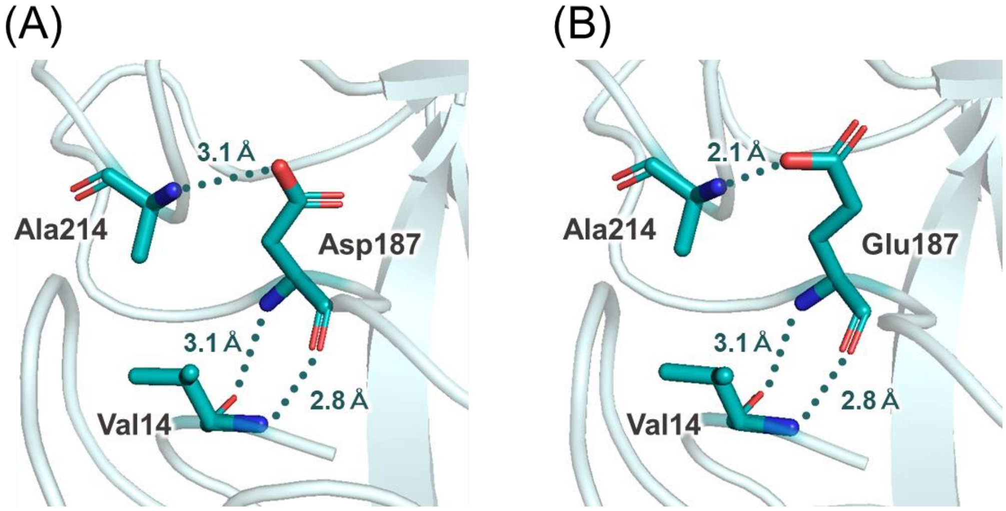

Figure S7. Schematic representation of the hydrogen bond network involving Asp187 (A) and Glu187 (B) in cocoonase. The Figure was produced using Pymol.

Table S1. Primer sequences used for PCR.

Primer name	Primer direction	Primer sequence	Mutation
pNS1	forward	5′ CTGTCAGACGACGAAGAGAAGATTGTC 3′	Lys8Asp
pNS1	reverse	5′ TTCGTCGTCTGACAGCGCTTCAGAAT 3′	Lys8Asp
pNS2	forward	5′ GTGGCAAAGAATCGTGTCAAGGCGAT 3′	Asp187Glu
pNS2	reverse	5′ GACACGATTCTTTGCCACCTTCCGG 3′	Asp187Glu
pNS3	forward	5′ CTGTCAGACGACGATGACAAGATTGTCGGTGGGGAA 3′	Lys8Asp, Glu10Asp, Glu11Asp
pNS3	reverse	5′ AATCTTGTCATCGTCGTCTGACAGCGCTTCAGAATC 3′	Lys8Asp, Glu10Asp, Glu11Asp
pNS4	forward	5′ GTGGCAAAGCATCGTGTCAAGGCGAT 3′	Asp187Ala
pNS4	reverse	5′ GACACGATTCTTTGCCACCTTCCGG 3′	Asp187Ala
pNS5	forward	5′ GTGGCAAAAACTCGTGTCAAGGCGAT 3′	Asp187Asn
pNS5	reverse	5′ GACACGAGTTTTTGCCACCTTCCGG 3′	Asp187Asn
pNS6	forward	5′ GTGGCAAATCTTCGTGTCAAGGCGAT 3′	Asp187Ser
pNS6	reverse	5′ GACACGAAGA TTTGCCACCTTCCGG 3′	Asp187Ser
pNS7	forward	5′ GCAAAGAAGACTGTCAAGGCGATTCAGG 3′	Asp187Glu, Ser188Asp
pNS7	reverse	5′ CTTGACAGTCTTCTTTGCCACCTTCCG 3′	Asp187Glu, Ser188Asp
pNS8	forward	5′ GCAAAGAAGAATGTCAAGGCGATTCAGG 3′	Asp187Glu, Ser188Glu
pNS8	reverse	5′ CTTGACATTCTTCTTTGCCACCTTCCG 3′	Asp187Glu, Ser188Glu
pNS9	forward	5′ CTGTCAGACGACGATGACAAGATTGTCGGTGGGGAA 3′	Lys8Asp, Glu10Asp, Glu11Asp
pNS9	reverse	5′ AATCTTGTCATCGTCGTCTGACAGCGCTTCAGAATC 3′	Lys8Asp, Glu10Asp, Glu11Asp
pNS10	forward	5′ GCAAAGACGACTGTCAAGGCGATTCAGG 3′	Ser188Asp
pNS10	reverse	5′ CTTGACAGTCGTCTTTGCCACCTTCCG 3′	Ser188Asp
pNS11	forward	5′ GCAAAGACGAATGTCAAGGCGATTCAGG3 ′	Ser188Glu
pNS11	reverse	5′ CTTGACATTCGTCTTTGCCACCTTCCG 3′	Ser188Glu
pNS13	forward	5′ GCAAAGACGCATGTCAAGGCGATTCAGG 3′	Ser188Ala
pNS13	reverse	5′ CTTGACATGCGTCTTTGCCACCTTCCG 3′	Ser188Ala
T_7	forward	5′ TAATACGACTCACTATAGG 3′	-
T_7	reverse	5′ CCCAAGGGGTTATGCTA 3′	-

Table S2. Expression vectors and abbreviations for mutant proteins.

Vector name	Mutant protein name	Abbreviation
pNS1	[K8D,K63G,K131G,K133A]-proCCN	[K8D]-proCCN′
pNS2	[K8D,D187E]-proCCN′	[K8D,D187E]-proCCN′
pNS3	[K8D,E10D,E11D,D187E]-proCCN′	cassette-[D187E]-proCCN′
pNS4	[K8D,E10D,E11D,D187A]-proCCN′	cassette-[D187A]-proCCN′
pNS5	[K8D,E10D,E11D,D187N]-proCCN′	cassette-[D187N]-proCCN′
pNS6	[K8D,E10D,E11D,D187S]-proCCN′	cassette-[D187S]-proCCN′
pNS7	[K8D,E10D,E11D,D187E,S188D]-proCCN′	cassette-[D187E,S188D]-proCCN′
pNS8	[K8D,E10D,E11D,D187E,S188E]-proCCN′	cassette-[D187E,S188E]-proCCN′
pNS9	[K8D,E10D,E11D]-proCCN′	cassette-proCCN′
pNS10	[K8D,E10D,E11D,S188D]-proCCN′	cassette-[S188D]-proCCN′
pNS11	[K8D,E10D,E11D,S188E]-proCCN′	cassette-[S188E]-proCCN′
pNS13	[K8D,E10D,E11D,S188A]-proCCN′	cassette-[S188A]-proCCN′

Table S3. Calculated volumes of the substrate binding pocket of CCN′ mutant proteins.

	Area (Å^2)	Volume (Å^3)	enzyme activity
Trpsin (PDB: 5T3H)	78.620	31.681	+++
CCN	76.701	33.711	+++
[K8D]-CCN'	75.688	33.528	+++
[D187E]-CCN'	75.918	34.486	++
[D187S]-CCN'	95.451	37.898	++
[D187A]-CCN'	101.986	40.689	+
[D187N]-CCN'	100.954	40.979	+
[S188A]-CCN'	83.304	37.223	+++
[S188D]-CCN'	72.286	29.349	+
[S188E]-CCN'	57.545	28.120	ND
[D187E,S188D]-CCN'	65.803	31.820	ND
[D187E,S188E]-CCN'	64.157	30.036	ND

+++: Strong, ++: weak, +: very weak, ND: not determined. The characteristic negative volumes were calculated using the CASTp program.
